# Blunted brachial blood flow velocity response to acute mental stress in PTSD females

**DOI:** 10.14814/phy2.16137

**Published:** 2024-07-05

**Authors:** Chowdhury Ibtida Tahmin, Chowdhury Tasnova Tahsin, Redeat Wattero, Zynab Ahmed, Chasity Corbin, Jason R. Carter, Jeanie Park, Susan B. Racette, Samaah S. Sullivan, Michael D. Herr, Ida T. Fonkoue

**Affiliations:** ^1^ Division of Physical Therapy and Rehabilitation Science, Department of Family Medicine and Community Health University of Minnesota Medical School Minneapolis Minnesota USA; ^2^ Robbins College of Health and Human Sciences Baylor University Waco Texas USA; ^3^ Division of Renal Medicine, Department of Medicine Emory University School of Medicine Atlanta Georgia USA; ^4^ Research Service Line, Atlanta VA Health Care System Decatur Georgia USA; ^5^ College of Health Solutions Arizona State University Phoenix Arizona USA; ^6^ Department of Epidemiology, Human Genetics, and Environmental Sciences, School of Public Health The University of Texas Health Science Center‐Houston Houston Texas USA; ^7^ Penn State Hershey Medical Center and Penn State College of Medicine Penn State University State College Pennsylvania USA

**Keywords:** blood flow velocity, endothelial dysfunction, females, PTSD, trauma

## Abstract

Post‐traumatic stress disorder (PTSD) is associated with increased cardiovascular disease (CVD) risk. Compared with males, females are twice as likely to develop PTSD after trauma exposure, and cardiovascular reactivity to stress is a known risk factor for CVD. We aimed to examine hemodynamic responses to acute mental stress in trauma‐exposed females with and without a clinical diagnosis of PTSD. We hypothesized that females with PTSD would have higher heart rate (HR), blood pressure (BP), and lower blood flow velocity (BFV) responsiveness compared with controls. We enrolled 21 females with PTSD and 21 trauma‐exposed controls. We continuously measured HR using a three‐lead electrocardiogram, BP using finger plethysmography, and brachial BFV using Doppler ultrasound. All variables were recorded during 10 min of supine rest, 5 min of mental arithmetic, and 5 min of recovery. Females with PTSD were older, and had higher BMI and higher resting diastolic BP. Accordingly, age, BMI, and diastolic BP were covariates for all repeated measures analyses. Females with PTSD had a blunted brachial BFV response to mental stress (time × group, *p* = 0.005) compared with controls, suggesting greater vasoconstriction. HR and BP responses were comparable. In conclusion, our results suggest early impairment of vascular function in premenopausal females with PTSD.

## 1 INTRODUCTION

Post‐traumatic stress disorder (PTSD) is a mental illness caused by repetitive and dysfunctional responses to trauma exposure (O'Donnell et al., 2021). Although the neurobiology of PTSD is not fully understood (Tolin & Foa, 2006), there are evidence of sex differences in PTSD development after exposure to similar traumatic events (Pooley et al., 2018). Females have a two to three times higher risk of developing PTSD compared with males. Furthermore, starting at a young age, females are more exposed to more interpersonal trauma such as sexual trauma (Olff, 2017; Olff et al., 2007). Finally, females diagnosed with PTSD are more prone than males to develop comorbidities such as cardiovascular disease (CVD) (Edmondson, 2017).

Psychological stress is a major contributor to CVD. Acute psychological stressors are responsible for increased blood pressure (BP) and heart rate (HR), while chronic psychological stressors can contribute to the development of atherosclerosis and hypertension (Huang et al., 2013). Available literature supports that chronic stress is associated with autonomic dysregulation (i.e., imbalance between sympathetic and parasympathetic activity) in individuals with PTSD (Thayer et al., 2010). This physiological biomarker of CVD has been observed in females with PTSD (Kibler, 2018). Prior studies have reported higher resting BP and HR caused by sympathetic overactivation in this population (Thayer et al., 2010; Yoo et al., 2020). Additionally, recent studies have revealed a possible vascular dysfunction in females with PTSD, as evidenced by higher arterial stiffness (Walczewska et al., 2011), an independent predictor of cardiovascular morbidity and mortality (Cecelja & Chowienczyk, 2012). It remains unclear whether vascular dysfunction could be the driver of CVD risk in young females with PTSD and if it is worsened during acute episodes of stress.

To date, there are no studies describing vascular responses to an acute psychological stressor in premenopausal females with a clinical diagnosis of PTSD. Our research group previously reported that that females aged 18–40 years and diagnosed with PTSD had higher resting arterial stiffness and blunted parasympathetic control of the heart than females in a trauma‐exposed control group (Ahmed et al., 2024). In the current study, we aim to expand on these findings to determine the extent to which young females diagnosed with PTSD will present with blunted brachial artery blood flow velocity (BFV), in response to acute mental stress compared with trauma‐exposed controls, indicative of greater vascular constriction and endothelial dysfunction. We hypothesized that females with PTSD would have a lower brachial BFV response to a mental arithmetic challenge (reflecting greater vasoconstriction and endothelial dysfunction), but higher BP and HR responses.

## 2 METHODS

### 2.1 Ethical overview and other considerations

All procedures in this study were approved by the Institutional Review Board of the University of Minnesota. This study only included young females. Therefore, the results may not be generalizable to other populations. However, given that young females are underrepresented in research and are increasingly at risk for PTSD and its related complications, including CVD, we believe the value of studying this population far outweighs any limitations of generalizability.

### 2.2 Study sample

Our study participants were recruited from the University of Minnesota Twin Cities and Saint Paul campus, surrounding communities, women's shelters through the University of Minnesota Medical Center, Fairview Hospital, and via referral. Inclusion criteria were premenopausal females aged 18–40 years with a history of trauma exposure, free of CVDs, and able to give their informed written consent for the study. Our exclusion criteria included trans women, men or non‐binary, as well as pregnant and breast‐feeding females. We also excluded those with comorbid conditions such as hypertension, dyslipidemia, heart disease, diabetes, vascular diseases such as Raynaud's phenomenon and Burgers's disease. Additional exclusion criteria included current illicit drug use, excessive alcohol use (>2 drinks per day), any autonomic dysfunction, any serious systemic disease such as systemic lupus erythematosus (SLE), medications for PTSD or other CVD, psychiatric comorbidities, severe traumatic brain injury, and the inability or unwillingness to abstain from nicotine use for at least 12 h before physiological testing. We assessed eligibility criteria via an online screening survey or phone interviews. Participants self‐reported their trauma exposure in writing, as part of our standardized measure for PTSD symptoms, described in Measures (the PTSD checklist for DSM‐5 [PCL‐5]).

### 2.3 Experimental protocol/design

Our study design was cross‐sectional. After completing the online screening survey, volunteers who met the inclusion criteria were invited to participate in two study visits. During the first study visit, we reviewed the consent form and explained study procedures. After providing written informed consent, participants completed surveys evaluating anxiety and depression symptoms severity.

We collected data on anthropometric measurements (height, weight, and abdominal circumference) and vitals. After 5–10 min of rest, we recorded three resting seated arterial BP and HR measurements using an appropriately sized automated BP cuff placed on the upper arm at the level of the heart (Omron, HEM 907XL, Omron Healthcare, Kyoto, Japan). Resting respiratory rate was obtained by visually counting respiratory chest movements. Before the second visit, which was scheduled 7 or more days after the first visit, participants were asked to abstain from heavy exercise, alcohol, and caffeine for 12 h prior to the visit. During this second visit (the “blood flow visit”), the volunteer rested for 10 min in the seated position and seated resting arterial BP and HR were recorded, as previously described. A three‐lead electrocardiogram (ECG) (ADInstruments Inc., Bio Amp FE231, Colorado Springs, Colorado, USA) was used for continuous recording of HR. Noninvasive arterial BP was measured using a servo‐controlled finger photoplethysmography (Human NIBP, ADInstruments Inc.), and BFV in the brachial artery was assessed via Doppler ultrasound (GE Versana Premier Louisville, KY). A Doppler audio translator (Herr et al., 2010) was used to transfer the Doppler signal from our ultrasound machine to our data acquisition system (PowerLab, ADInstruments, NSW, Australia). This allowed us to record BFV continuously while simultaneously recording all other hemodynamics variables. After obtaining good quality signals in each variable measured, we recorded 10 min of baseline, 5 min of mental stress (achieved via mental arithmetic stress task), and 5 min of recovery. The mental arithmetic stress task consisted of serial subtraction of a single‐digit number from double‐ and triple‐digits numbers, as fast as the participant could under pressure. A rated perceived stress score with an ordinal scale of 1–4 was recorded after mental stress, with “1” referring to “not stressful and 4” referring to “very stressful.” Mental stress is a universal experience in our lives (Sara et al., 2022) and is a well‐known laboratory stressor to assess cardiovascular dysfunction (Vancheri et al., 2022).

### 2.4 Measures

#### 2.4.1 Exposure variables: PTSD Checklist for DSM‐5 with criterion a (traumatic event)

The PCL‐5 is a 20‐item self‐reported questionnaire based on the fifth edition of the Diagnostic and Statistical Manual of Mental Disorders (DSM‐5) symptoms of PTSD (Bovin et al., 2016), one of the most widely used screening measures for PTSD. The PCL‐5 total score ranges from 0 to 80, with a score between 31 and 33 considered as probable PTSD, a score between 33 and 45 considered mild, scores between 45 and 60 considered moderate, and a score of more than 60 classified as severe PTSD. In this study, the PCL‐5 questionnaire was only used to evaluate the severity of symptoms and not for diagnosis. Our participants with PTSD were enrolled in our study based on their prior clinical diagnosis.

### 2.5 Outcome variables

#### 2.5.1 Brachial artery blood flow velocity

We quantified brachial artery BFV as our primary outcome variable. We used GE Versana premier Doppler ultrasound as the standard imaging platform in our study for the visualization of brachial artery blood flow. Participants laid supine on a stretcher bed with their right arm comfortably supported on a side table at the level of their heart. A 12 L 10 MHz linear array probe of the Doppler ultrasound at an angle of 60° was used. To obtain the best view of the brachial artery and a high‐quality sound of brachial artery blood flow, we used a Doppler audio translator (DAT) (Herr et al., 2010) to directly convert Doppler ultrasound audio signals into real time fluid flow velocity analogue signals. The Doppler audio signals were integrated into the LabChart physiological data analysis software, continuously and simultaneously with our other outcome variables (Herr et al., 2010). Lower values recorded during measurement of BFV of the brachial artery are indicative of greater vasoconstriction and transient endothelial dysfunction. Of note, all recorded data were captured via LabChart. Given that data were continuously recorded for 10 min of baseline, 5 min of mental stress, and 5 min of recovery, no data were stored in the Doppler ultrasound device. BFV is used in this study as a surrogate marker of endothelial function (Kashyap et al., 2013; Minhas et al., 2022), allowing us to continuously measure vascular changes in response to a mental stress task, simultaneously with beat‐to‐beat changes in BP and HR.

#### 2.5.2 HR, BP, and respiratory rate

Seated resting arterial BP and HR were measured three times at 1‐min intervals, according to the standard American College of Cardiology and American Heart Association guidelines (Whelton et al., 2018). In addition, we measured BP and HR after 10 min of quiet rest using an automated digital BP device (Omron, HEM‐907XL, Omron Healthcare, Kyoto, Japan), with an appropriately sized cuff placed on the upper arm and the arm resting at heart level. During our experimental protocol, a noninvasive finger cuff device (Human NIBP NANO INL 382 FINAPRES MEDICAL SYSTEM) with an appropriately sized finger cuff measured beat‐to‐beat arterial BP for each participant. From the upper arm BP recording, we calibrated absolute values of BP by using an internal calibration system (Fonkoue et al., 2023). HR was measured by the 3‐lead ECG with a Bio Amp placed in appropriate angle with the heart (model ML 132, AD Instrument, Colorado Springs, CO). Additionally, we used a respiratory belt transducer to measure the respiratory waveforms (AD Instruments TN1132/ST). All of these measures were recorded simultaneously with the BFV by Doppler audio translator (Herr et al., 2010).

#### 2.5.3 Data and statistical analysis

Data were processed using the software LabChart. We used this data analysis software to quantify the mean, maximum, and minimum of our main outcome variable, BFV. BP (systolic, diastolic, and mean arterial pressure) and HR are calculated and reported as the mean of each time point. The three time points analyzed were the last 5 min of baseline, 5 min of mental arithmetic, and 5 min of recovery.

For statistical analyses, we used the data analysis software SPSS v.28. We categorized our participants into PTSD and non‐PTSD groups based on their self‐reported clinical diagnosis of PTSD. We used independent samples *t*‐tests to compare baseline demographics, such as age, BMI, PCL‐5, resting BP, and HR. The response to stress (absolute change from baseline to mental arithmetic) and recovery from stress (changes in relation to baseline values) to stress were analyzed for both groups using repeated measure ANCOVA, with time point as within factor and PTSD diagnosis as between factor. Significance level was set at *p* < 0.05.

## 3 RESULTS

### 3.1 Baseline demographics and resting hemodynamics

A total of 42 trauma‐exposed premenopausal females from diverse backgrounds were enrolled in the study. Twenty‐one participants were categorized as PTSD (clinically diagnosed with PTSD) and 21 as controls (history of trauma exposure but not diagnosed with PTSD). Of note, three controls with PCL‐5 scores >40 were recategorized as PTSD.

Baseline characteristics are shown in Table 1 for both groups. Females with PTSD were older (*p* = 0.022), and had higher BMI (*p* = 0.013) and higher resting diastolic BP (*p* = 0.013) compared with females without PTSD (controls). Resting systolic BP was comparable between the females with PTSD and controls (*p* = 0.386). Resting MAP tended to be higher in PTSD compared with controls (*p* = 0.056). Resting HR was also similar between females with PTSD and controls (*p* = 0.470). Resting respiratory rates were marginally higher among females with PTSD (*p* = 0.066). As expected, PCL‐5 scores also were significantly higher among females with PTSD compared with controls (*p* < 0.001).

**TABLE 1:** Descriptive characteristics.

	PTSD (*n* = 21)	Controls (*n* = 21)	*p*‐value
Race (%) (Whites/Blacks/others)	(57/38/5)	(57/19/24)	—
Age (years)	30 ± 9	25 ± 5	0.022
BMI (kg/m^2^)	29.4 ± 5.8	25.4 ± 4.3	0.013
Contraceptive use (%) (yes/no)	(52/48)	(71/29)	—
Tobacco use (%) (yes/no)	(19/81)	(19/81)	—
PCL‐5 (a.u.)	49 ± 13	22 ± 12	<0.001
Resting SBP (mmHg)	108 ± 13	105 ± 11	0.386
Resting DBP (mmHg)	72 ± 8	66 ± 9	0.013
Resting MAP (mmHg)	84 ± 9	79 ± 9	0.056
Resting HR (beats/min)	76 ± 12	74 ± 9	0.470
Resting RR (breaths/min)	16 ± 3	15 ± 3	0.066
Resting mean BFV (cm/s)	4.6 ± 2.25	3.6 ± 1.7	0.123

*Note*: Values are presented as mean ± SD. “Others” for race included Asian, Hispanic/Latino, and American Indian. All *p*‐values are two‐tailed.

Abbreviations: BFV, blood flow velocity; BMI, body mass index; DBP, diastolic blood pressure; HR, heart rate; MAP, mean arterial pressure; PCL‐5, PTSD checklist for DSM‐5 criterion A; RR, respiratory rate; SBP, systolic blood pressure.

Given the differences in baseline demographics and resting hemodynamics, we controlled for age, BMI, and diastolic BP as a covariate in all repeated measures analyses. Time (baseline, mental stress, and recovery) was the within‐subject factor and group (PTSD and control) was the between‐subject factor. In a secondary analysis, we controlled for contraceptives' use. Results presented below are mean BFV, mean BP, and mean HR. The symbol “Δ” represents the change in a variable from baseline.

### 3.2 Effects of mental stress on brachial BFV

**FIGURE 1:**
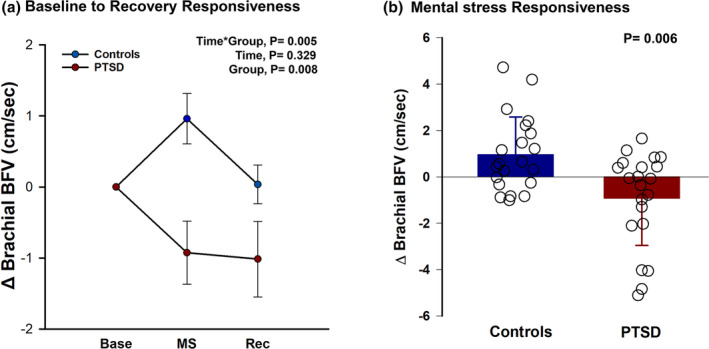
(a) Changes (Δ) in brachial blood flow velocity (BFV), from baseline (Base) to mental stress (MS) and to recovery (Rec), between 21 trauma‐exposed females without PTSD (controls) and 21 women clinically diagnosed with PTSD. We used repeated measures ANCOVA with study timepoint (time) as within factor and PTSD diagnosis (group) as between factor to compare the reactivity and recovery to acute mental arithmetic between the two groups. Women with PTSD had a blunted brachial BFV response from baseline to recovery compared with controls. (b) Brachial BFV reactivity (mean and individual responses) at the mental stress (MS) timepoint of (a), between controls and PTSD. We ran an independent *t*‐test to compare Δ BFV between the two groups. Women with PTSD had a blunted brachial BFV reactivity to the mental arithmetic task compared with controls.

We assessed the response of BFV to the acute stress protocol. Figure 1 depicts the changes in mean brachial BFV between the two groups. Females with PTSD had a blunted brachial BFV response to mental stress (time × group, *p* = 0.005, *ηp* ^2^ = 0.137) when compared with controls (Figure 1a). Specifically, post hoc comparisons revealed that in response to mental arithmetic (Figure 1b), mean BFV decreased in PTSD (Δ = −0.92 ± 2.03 cm/s) but increased in controls (Δ = +0.96 ± 1.62 cm/s, *p* = 0.006 between groups). Furthermore, mean BFV continued to decrease during recovery (result not graphed) in PTSD (Δ = −1.02 ± 2.43), suggesting vasoconstriction but remained slightly higher than baseline in controls (Δ = +0.04 ± 1.25 cm/s, *p* = 0.044). The results remained unchanged when we added contraceptives' use to the list of covariates (time × group, *p* = 0.014) or when we analyzed the subset of 15 PTSD and 20 controls (time × group, *p* = 0.010) who were matched for age (28 ± 7 vs. 25 ± 5 years, *p* = 0.264) and BMI (27.8 ± 4.3 vs. 25.8 ± 4.0 kg/m^2^, *p* = 0.174), respectively.

### 3.3 HR and BP responses to acute mental stress

**FIGURE 2:**
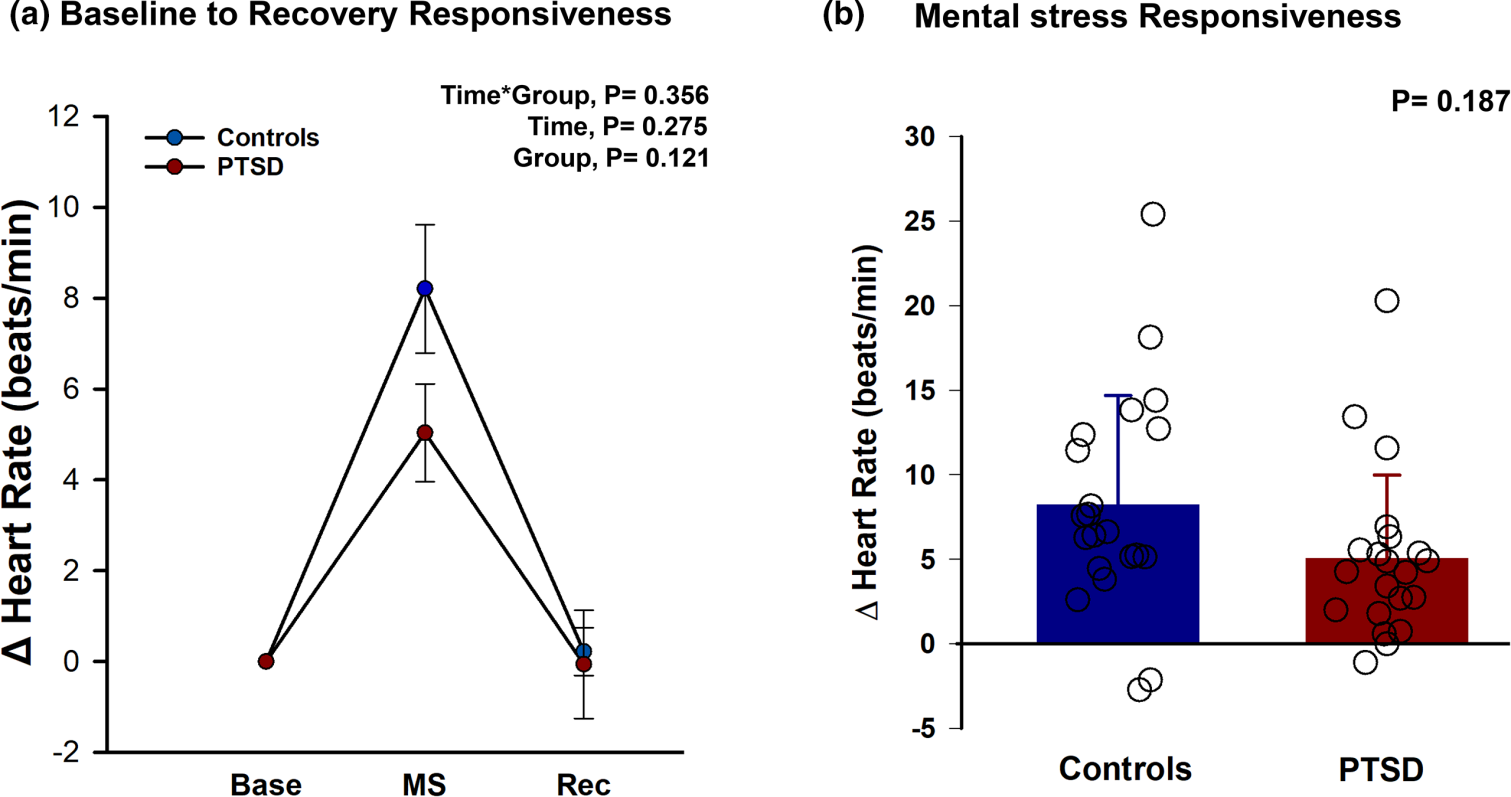
(a) Changes (Δ) in heart rate, from baseline (Base) to mental stress (MS) and to recovery (Rec), between 21 trauma‐exposed females without PTSD (controls) and 21 women clinically diagnosed with PTSD. We used repeated measures ANCOVA with study timepoint (time) as within factor and PTSD diagnosis (group) as between factor to compare the reactivity and recovery to acute mental arithmetic between the two groups. Heart rate response was comparable from baseline to recovery between controls and PTSD. (b) Heart rate reactivity (mean and individual responses) at the mental stress (MS) timepoint of (a), between controls and PTSD. We ran an independent *t*‐test to compare Δ HR between two groups. In response to the mental arithmetic task, heart rate similarly increased for both controls and PTSD.

In response to the acute stress protocol, HR responses (Figure 2a) were comparable for PTSD and controls (time × group = 0.356, *ηp* ^2^ = 0.027). Specifically, during the mental arithmetic stress task (Figure 2b), mean HR increased similarly in PTSD and controls (Δ = +5.04 ± 5 and +8.21 ± 6.5 beats/min, respectively, *p* = 0.187). Heart rate during recovery (result not graphed) was also similar for PTSD and controls (Δ = −0.1 ± 5.5 and +0.22 ± 2.4 beats/min, respectively, *p* = 0.262). The results remained comparable (time × group, *p* > 0.05) when we analyzed the subset of 15 PTSD and 20 controls matched for age and BMI.

**FIGURE 3:**
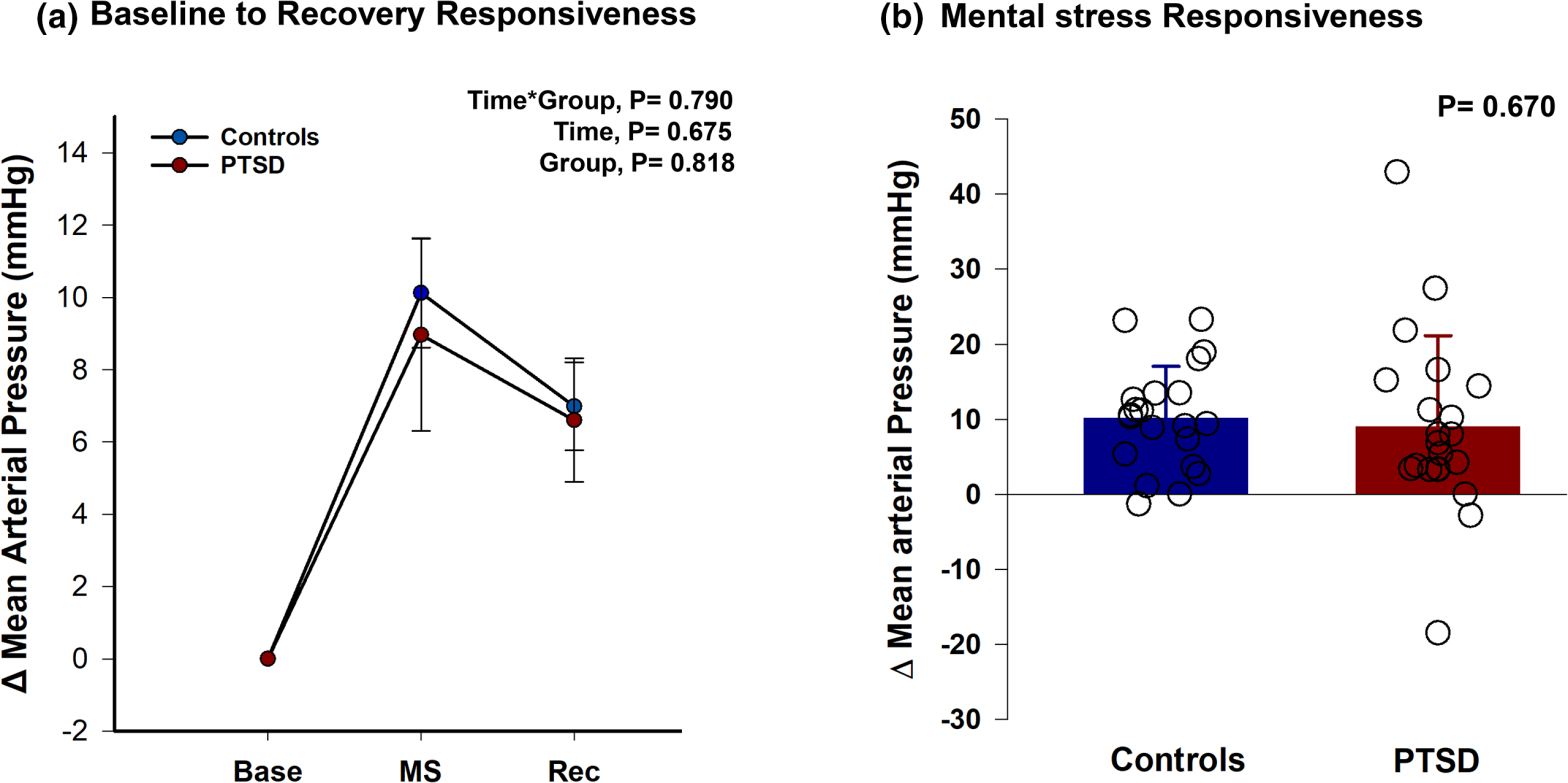
(a) Changes (Δ) in mean arterial pressure, from baseline (Base) to mental stress (MS) and to recovery (Rec), between 21 trauma‐exposed females without PTSD (controls) and 21 women clinically diagnosed with PTSD. We used repeated measures ANCOVA with study timepoint (time) as within factor and PTSD diagnosis (group) as between factor to compare the reactivity and recovery to acute mental arithmetic between the two groups. Mean arterial pressure response was comparable from baseline to recovery between controls and PTSD. (b) Mean arterial pressure reactivity (mean and individual responses) at the mental stress (MS) timepoint of (a), between controls and PTSD. We ran an independent *t*‐test to compare Δ mean arterial pressure between the two groups. In response to the mental arithmetic task, mean arterial pressure similarly increased for both controls and PTSD.

Likewise, mean arterial pressure (Figure 3a) response was not significantly different between the two groups (time × group, *p* = 0.790, *ηp* ^2^ = 0.006). In response to mental arithmetic (Figure 3b), mean arterial pressure also increased in both PTSD (9 ± 12.17) and control (10.12 ± 7 mmHg, *p* = 0.670), and during recovery (result not graphed), mean arterial pressure was also comparable in PTSD (6.60 ± 8) and control (7 ± 6 mmHg, *p* = 0.927). The results remained comparable (time × group, *p* > 0.05) when we analyzed the subset of 15 PTSD and 20 controls and matched for age and BMI.

Rated perceived stress was comparable between controls and PTSD (2.9 ± 0.6 vs. 3.0 ± 0.6, *p* = 0.408, respectively).

For all analyses, the results were unchanged when we replaced diastolic BP with MAP as a covariate.

Our ANCOVA results remained comparable even when the three controls recategorized as PTSD were excluded from the analysis [(BFV, time × group, *p* = 0.014, *ηp* ^2^ = 0.118), (HR, time × group, *p* = 0.468, *ηp* ^2^ = 0.021) and (MAP, Time × group, *p* = 0.846, *ηp* ^2^ = 0.005)]. The *p* values are two‐tailed.

## 4 DISCUSSION

In this pilot study, we found that females clinically diagnosed with PTSD have lower brachial BFV reactivity and recovery to a mental arithmetic stress task compared with a control group of trauma‐exposed females without a PTSD diagnosis. These results indicate that females with PTSD have greater vasoconstriction of the brachial artery during mental stress, which is indicative of greater endothelial dysfunction. However, HR and BP responses were similar between the two groups of females from baseline to recovery. Importantly, although premenopausal females are often thought to be more protected from CVD, our results show that young females with PTSD display abnormal vascular responses to acute mental stress, which could be indicative of future risk of cardiovascular events. Our findings highlight and further support the association of PTSD and vascular dysfunction in the etiology of CVD.

Sympathetic stimulation in response to mental stress is known to have paradoxical effects on the vasculature (Harris et al., 2000). In healthy vessels, mental stress causes the release of norepinephrine which binds to ⍺1 receptors, stimulates smooth muscles, and results in vasoconstriction (Froese et al., 2020; Harris et al., 2000). On the other hand, mental stress also causes a rise in circulating epinephrine, leading to β2‐mediated vasodilation by increasing nitric oxide bioavailability, which is responsible for endothelium‐dependent vasodilation (Carter et al., 2005; Conti et al., 2013; García De Lomana et al., 2022; Joyner & Casey, 2009). The vascular endothelium has an important role in the control of vascular tone (Furchgott & Zawadzki, 1980; Sandoo et al., 2010). Studies have shown that shear stress, which results from increases in blood flow, causes an endothelium‐dependent vasodilator response of the blood vessels (Paniagua et al., 2001; Pyke & Tschakovsky, 2005). This vasodilatory response stems from the stimulation of vascular mechanoreceptors and the subsequent release of nitric oxide, a powerful vasodilator (Harris et al., 2000). Furthermore, nitric oxide can directly inhibit the vasoconstrictor effect of catecholamines on vascular smooth muscles (Conti et al., 2013; Joyner & Casey, 2009). In sum, there is strong evidence that nitric oxide is a major contributor to the vasodilator response to mental stress in healthy blood vessels. Given that several studies have supported a link between chronic psychosocial stress and increased risk of CVD (Henein et al., 2022; Poitras & Pyke, 2013), endothelial dysfunction might be an early underlying mechanism to consider.

In our current study, we investigated the effects of acute mental stress on brachial BFV during a mental arithmetic stress task and during recovery after the stress in premenopausal females with PTSD. We found a blunted BFV reactivity to acute stress and recovery from stress in the PTSD group, suggesting persistent vasoconstriction. As aforementioned, studies have shown that the release of nitric oxide during mental stress causes vasodilation (Joyner & Casey, 2009) and thereby increasing BFV (Harris et al., 2000; Matienzo & Bordoni, n.d.). Harris et al. highlights that a decrease in the radius of a blood vessel (i.e., vasoconstriction) leads to reduced blood flow (Harris et al., 2000), and additionally, Matienzo et al. reported reduced BFV by reducing blood flow (Matienzo & Bordoni, 2023). A decrease in flow‐mediated dilation during mental stress has been attributed to a disruption in nitric oxide activity, resulting in an inability of the endothelium to offset norepinephrine‐induced vasoconstriction (Lima et al., 2019). Thus, the lack of vasodilatory response to mental arithmetic in premenopausal females with PTSD in our study suggests a possible impairment of the endothelium‐dependent vasodilation. In contrast, in our control group, we observed the expected vasodilatory response during stress.

Studies investigating flow‐mediated dilation response to mental stress in other patient populations have reported mixed results. Wagner et al found a decreased response in participants with depression, while D'Urzoa et al. reported no difference (D'Urzo et al., 2019; Wagner et al., 2012). Studies in female and male patients with pre‐existing coronary artery disease have linked transient endothelial dysfunction observed during mental stress to major adverse cardiovascular events (Ghiadoni et al., 2000; Lima et al., 2019; Vaccarino et al., 2021). Another study of patients with underlying ischemic heart disease found that endothelial dysfunction of the coronary artery was associated with major adverse cardiovascular events in both males and females, but microvascular dysfunction measured via reactive hyperemia index was only associated with adverse events among females (Sullivan et al., 2023).

In contrast to our BFV results, BP and HR responses to mental arithmetic were comparable between groups in the present study. Acute mental stress has been known to increase HR and BP in health (Formolo et al., 2022) and in disease conditions (Formolo et al., 2022; Vancheri et al., 2022). The cardiovascular reactivity hypothesis posits that individuals with an exaggerated HR and BP reactivity to a laboratory stressor such as a mental arithmetic stress task have a higher risk of developing hypertension later in life (Ginty et al., 2022; Yuenyongchaiwat, 2017). For that reason, we hypothesized that females with PTSD would have higher HR and BP responses to mental stress compared with trauma‐exposed females without PTSD. However, our results do not support this hypothesis. It has been suggested that frequent exposure to an array of psychological stressors such as frequent reminders of trauma (triggers) that cause PTSD might lead to a form of psychological and/or physiological adaptation that inhibits certain arousal responses, such as sympathetic activation during an acute laboratory‐based mental stress (Fonkoue et al., 2018). This may explain why females with PTSD in the present study did not have a greater HR and BP response to mental stress, as originally hypothesized. Finally, it is possible that within the control group, the HR increase in response to sympathetic stimulation led to an increase in cardiac output (and eventually increase in blood flow and blood flow velocity) which might be driving the BP response. In contrast, in females with PTSD, there was an increase HR and BP while BFV was decreasing. This might suggest that increases in vascular resistance, causing persistent vasoconstriction, might be the driver of the comparable increase in BP observed in the PTSD group.

### 4.1 Limitations

There were some limitations to our study. Our sample size was relatively small and included only premenopausal females. Therefore, the results of this study may not be generalizable to older females or males. However, given that females who have been exposed to trauma and developed PTSD have been understudied, we believe that this study has clinical importance. Second, we did not control for generations of hormonal contraceptives, which may have differentially impacted our outcome measures. Future studies may need to include a larger sample size to account for generations of exogenous hormones. Shenouda et al. revealed that although brachial endothelial function was not affected by hormonal changes during the menstrual cycle in premenopausal females, it was negatively correlated with long‐term use of second‐generation oral contraceptive pills (Shenouda et al., 2018). Third, unlike in previous studies (Cardillo et al., 1997; Dietz et al., 1994; Limberg et al., 2010; Schrage et al., 2007), we did not capture ultrasound images to assess brachial artery diameter for this study. Our aim was to obtain continuous recording of blood flow velocity before, during, and after a mental arithmetic challenge. We used a Doppler audio translator to continuously integrate all data into LabChart and failed to store data in the Doppler ultrasound device. Thus, we are unable to exclude that the change in the velocity could have resulted from a change in the diameter or from a change in mean BP.

### 4.2 Strengths

The major strength of this study was the simultaneous measurement of continuous BFV, BP, and HR at rest, during mental stress, and during recovery using the same temporal alignment, in the same data acquisition system, within PTSD females and controls. The second strength of our study is the fact that all participants had a history of trauma exposure. Thus, our findings shed light on the added burden of a PTSD diagnosis beyond trauma.

## 5 CONCLUSIONS

In the present study, mental stress was associated with possible vascular dysfunction in PTSD females compared with trauma‐exposed controls, as evidenced by decreased brachial BFV. In contrast, there were no significant differences in HR and BP responses to stress between females with and without PTSD. Results from this pilot study may help to elucidate a mechanistic pathway linking PTSD to CVD in females, prior to menopause.

## FUNDING INFORMATION

This study was supported by the following grants: K01HL161027, UMN CTSI UL1TR002494, and U54AT012307.

## ETHICS STATEMENT

This study was approved by the Institutional Review Board of the University of Minnesota. Informed consent was obtained from all participants, and all procedures and protocols conformed with the standards of use of human participants in research as outlined in the Sixth Declaration of Helsinki.

## Data Availability

Data used for this study will be available upon request.
